# The Baltic Sea Virome: Diversity and Transcriptional Activity of DNA and RNA Viruses

**DOI:** 10.1128/mSystems.00125-16

**Published:** 2017-02-14

**Authors:** Lisa Zeigler Allen, John P. McCrow, Karolina Ininbergs, Christopher L. Dupont, Jonathan H. Badger, Jeffery M. Hoffman, Martin Ekman, Andrew E. Allen, Birgitta Bergman, J. Craig Venter

**Affiliations:** a Microbial and Environmental Genomics, J. Craig Venter Institute, San Diego, California, USA; b Scripps Institution of Oceanography, La Jolla, California, USA; c Department of Ecology, Environment and Plant Sciences, Stockholm University/Science for Life Laboratory, Solna, Sweden; Georgia Tech

**Keywords:** marine microbiology, viral ecology, viral metagenomics, viral metatranscriptomics, viral/host inference

## Abstract

Metagenomic and metatranscriptomic data were generated from size-fractionated samples from 11 sites within the Baltic Sea and adjacent marine waters of Kattegat and freshwater Lake Torneträsk in order to investigate the diversity, distribution, and transcriptional activity of virioplankton. Such a transect, spanning a salinity gradient from freshwater to the open sea, facilitated a broad genome-enabled investigation of natural as well as impacted aspects of Baltic Sea viral communities. Taxonomic signatures representative of phages within the widely distributed order *Caudovirales* were identified with enrichments in lesser-known families such as *Podoviridae* and *Siphoviridae*. The distribution of phage reported to infect diverse and ubiquitous heterotrophic bacteria (SAR11 clades) and cyanobacteria (*Synechococcus* sp.) displayed population-level shifts in diversity. Samples from higher-salinity conditions (>14 practical salinity units [PSU]) had increased abundances of viruses for picoeukaryotes, i.e., *Ostreococcus*. These data, combined with host diversity estimates, suggest viral modulation of diversity on the whole-community scale, as well as in specific prokaryotic and eukaryotic lineages. RNA libraries revealed single-stranded DNA (ssDNA) and RNA viral populations throughout the Baltic Sea, with ssDNA phage highly represented in Lake Torneträsk. Further, our data suggest relatively high transcriptional activity of fish viruses within diverse families known to have broad host ranges, such as *Nodoviridae* (RNA), *Iridoviridae* (DNA), and predicted zoonotic viruses that can cause ecological and economic damage as well as impact human health.

**IMPORTANCE** Inferred virus-host relationships, community structures of ubiquitous ecologically relevant groups, and identification of transcriptionally active populations have been achieved with our Baltic Sea study. Further, these data, highlighting the transcriptional activity of viruses, represent one of the more powerful uses of omics concerning ecosystem health. The use of omics-related data to assess ecosystem health holds great promise for rapid and relatively inexpensive determination of perturbations and risk, explicitly with regard to viral assemblages, as no single marker gene is suitable for widespread taxonomic coverage.

## Abstract (precis)

Inferred virus-host relationships, community structures of ubiquitous ecologically relevant groups, and identification of transcriptionally active populations have been achieved with our Baltic Sea study. Further, these data, highlighting the transcriptional activity of viruses, represent one of the more powerful uses of omics concerning ecosystem health. The use of omics-related data to assess ecosystem health holds great promise for rapid and relatively inexpensive determination of perturbations and risk, explicitly with regard to viral assemblages, as no single marker gene is suitable for widespread taxonomic coverage.

## INTRODUCTION

Viruses are ubiquitous in the world’s oceans (10^10^ per liter [1]), are a vast source of genetic diversity, and play an important role in biogeochemical processes. Here we refer to viruses collectively, independently of host or mode of infection. Generally, viruses are small and their genomes encode relatively few proteins, and yet despite this deceptive simplicity, the diversity of mechanisms used for replication and biochemistry is unrivaled in cellular counterparts. Viruses persist by exploiting the vital interactions with their host necessary to complete replication. Hosts may be of bacterial, eukaryotic, archaeal, or even viral (e.g., *Mamavirus*) origin. Within the oceans, the marine microbial food web plays a major role in the recycling of carbon and nutrients and in regulating energy transfer to higher trophic levels (2, 3). Viruses play an essential role in fueling this loop through cell lysis of bacteria and phytoplankton, thus triggering cellular release of significant amounts of fixed carbon and nitrogen associated with dissolved organic matter (DOM) (1). This viral shunt (4, 5) of the microbial loop represents a major source of DOM for bacterial consumption and increases the level of CO_2_ respiration of the entire ecosystem.

The Baltic Sea area is typified by a distinct north-to-south transition from freshwater to marine salinity levels and various nutrient regimens, the latter largely resulting from anthropogenic sources causing eutrophication. The Baltic Sea represents an exceptionally dynamic environment for biota, with a long residence time of 25 to 30 years, high levels of freshwater inputs originating largely from river runoff (~2%/volume) relative to precipitation and evaporation (6), and extreme seasonal temperature variations (−0.3 to 20°C).

Metagenomic investigations within marine environments started in the early 2000s with pioneering efforts leading to the discovery of dominant taxa and vital functions that had not been previously identified (7, 8). While numerous marine virome studies have also been reported, most studies predominantly targeted double-stranded DNA (dsDNA) genomes using traditional methods (9–12) and therefore conveyed only an abridged understanding of marine viromes. Global DNA sequence-based analyses of the Baltic Sea have been developed more recently, with the first high-throughput 16S rRNA gene assessment of bacterial seasonality performed in 2010 (13–15), although previous low-throughput studies of molecular markers have been reported. Since that time, there have been numerous signature gene studies (see, e.g., references 16, 17, and 18), three metagenomics studies (16, 19–22), and one metatranscriptomics study (23, 24). Fewer investigations have focused on Baltic Sea viruses, particularly those infecting ecologically relevant groups, including *Cyanobacteria*, *Proteobacteria*, and eukaryotic algae. Recently, Šulčius and Holmfeldt reviewed the status of viral research in the Baltic Sea and pointed to the significance of bacteriophage, particularly the most studied phages of the *Bacteriodetes* phylum (25). The anoxic waters of the Baltic Sea were found to have high levels of virally induced microbial mortality (26), and viral production was inversely correlated with total phosphorus levels, particularly in the Bothnian Bay region, in a more recent study (27, 28). Further, characterization of phages, including the isolation (29) and genomic variability (30) of bacteriophages from sea ice as well as the morphologies of sediment phages (31), has been reported. Nevertheless, the genomic characterization of viruses in the Baltic Sea has greatly lagged behind that of their prokaryotic and eukaryotic hosts.

**FIG 1:**
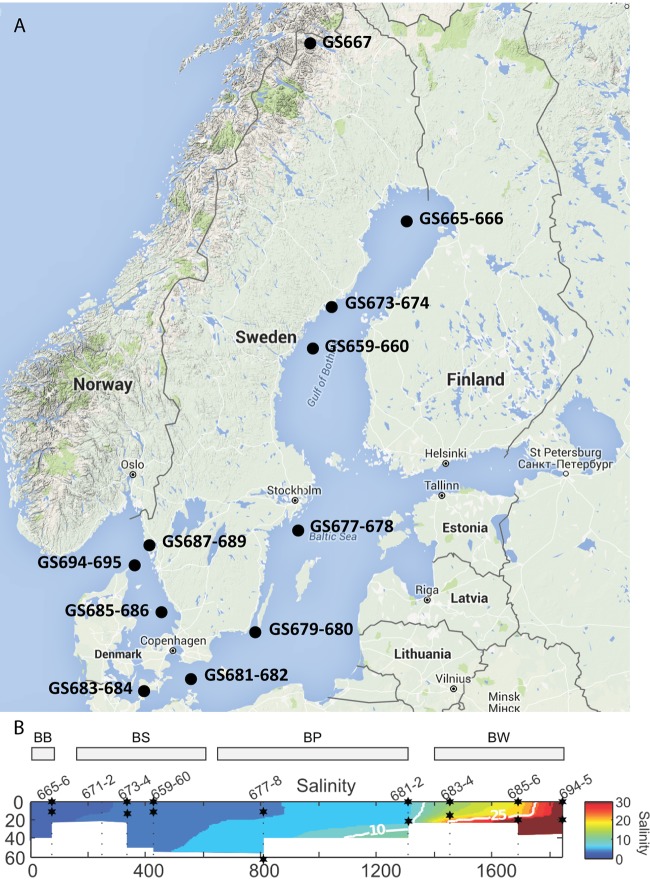
Baltic Sea maps. (A) Sites and identifiers shown traversing the Baltic Sea (BB, Bothnian Bay; BS, Bothnian Sea; BP, Baltic Proper; BW, Baltic West). (B) Salinity and depth profiles of samples with virus size libraries (0.1 µm to 50 kDa) are in red; data were adapted with permission from Dupont et al. (19). Note Lake Torne Träsk in the north of Sweden.

Here we report the first targeted study of natural and human-impacted viral representation in the Baltic Sea and one nearby freshwater lake using modern genome-enabled technologies. Metagenomic libraries of samples from 21 sites, 7 consisting of virus-size-specific libraries (0.1 µm to 50 kDa), were combined with 39 metatranscriptomic (transcriptionally active; mRNA) libraries from 13 sites comprising multiple microbial size classes (Fig. 1). Analyses of coupled metagenomics and metatranscriptomics data provide the first detailed picture of spatial variations in viral diversity and transcriptional activity within the Baltic Sea ecosystem.

## RESULTS

### Sampling scheme.

The Baltic Sea is the world’s second largest body of brackish water, and the microbial communities present have been intensively studied for biogeographic patterns, diversity estimates, dynamics, and ecological significance. Recently, our team published a thorough metagenomic analysis of the microbial consortia and described functional attributes and community composition traversing the salinity gradient, with a primary emphasis on the prokaryotic community (19). Here we utilize this data set as well as additional virus size-class samples and metatranscriptomic data to examine the viral consortia. The samples represent a salinity gradient of 0 to 34.35 practical salinity units (PSU) and include a freshwater Arctic lake, Lake Torneträsk (Abisko, Sweden; site GS667) (Fig. 1 and Table S1 in the supplemental material). Sample sites span the following subbasins within the Baltic Sea area: Bothnian Bay (BB), Bothnian Sea (BS), Baltic Proper (BP), and Baltic West (BW [referring to sites off the Swedish west coast within Kattegat and Skagerrak Bays connecting to the Arctic North Sea). In total, approximately 23 million and 8.6 million predicted peptides from the metagenomic and metatranscriptomic data sets, respectively, were analyzed in this study (Table S1B and C). To compare the virus size-class samples to those from previous Global Ocean Sampling (GOS) expeditions, orthology and similarity analyses were used on all predicted protein sequences, including those from samples collected from (i) the Baltic Sea, (ii) the Indian Ocean (9), (iii) Chesapeake Bay, and (iv) the California Current. As expected, the majority (51.3%) of sequences are similar to those of samples from Chesapeake Bay, also a brackish system with eutrophication as a major perturbation, and from the California Current, which were similar to the BW samples, while 10.5% were unique to the Baltic Sea area (see Fig. S2 in the supplemental material).

10.1128/mSystems.00125-16.8 TABLE S1  Oceanographic contextual data and sequence statistics for samples of DNA and RNA libraries. Download TABLE S1, XLSX file, 0.04 MB. Copyright © 2017 Zeigler Allen et al. 2017 Zeigler Allen et al. This content is distributed under the terms of the Creative Commons Attribution 4.0 International license.

### Virome diversity.

Using metagenomic data, we identified the taxonomic diversity of major natural dsDNA viral groups; not surprisingly, taxonomic distributions differed between the viral sequences obtained from cell-sized filters (200 to 0.1 μm) and those from the virus-sized fraction (0.1 μm to 50 kDa), possibly indicative of viruses associated with hosts and ambient viruses that were detected between infection cycles, respectively (Fig. S1). The largest group of viruses consisted of members of the *Caudovirales* (tailed phages), which include the families *Myoviridae* (with contractile tails), *Siphoviridae* (with noncontractile tails), and *Podoviridae* (with short tails). These bacteriophages infect a wide range of microbial hosts, many of which are dominant phyla in marine environments, including *Cyanobacteria*, *Proteobacteria*, and *Bacteroidetes*. *Siphoviridae* viruses were not detected at appreciable levels in the open reading frame (ORF)-based metagenomic analysis. Interestingly, sequences phylogenetically related to N4-like viruses were present within the virus-size and larger fractions for sample GS678 within the Landsort Deep. The species represented was *Enterobacteria* phage N4; however, the database did not contain more recent N4-like isolate genomes (e.g., *Roseophage* genomes) that might be more relevant for marine environments.

10.1128/mSystems.00125-16.1 FIG S1  Taxonomic bins of viral sequences within virus-size fractions (0.1 μm to 50 kDa) (A) and fractions of larger sizes (200 to 0.1 μm) (B). Sequences similar to those of N4-like viruses were displaced from the *Podoviridae* group and are shown in green. Download FIG S1, JPG file, 0.1 MB. Copyright © 2017 Zeigler Allen et al. 2017 Zeigler Allen et al. This content is distributed under the terms of the Creative Commons Attribution 4.0 International license.

10.1128/mSystems.00125-16.2 FIG S2  Comparison of Baltic Sea virome data sets (Baltic) to other GOS virome data sets from the California Current (Cal), Cheasepeake Bay (CBay), and Indian Ocean (IO). (A and B) Comparisons performed using similarity of sequences (A) and using orthology (B). (C) Number of predicted orthologous peptides for each data set following similarity searches against other bins to reduce to number of purely unique peptides. Definitions of similarity and orthology are given in the box (inset) for each diagram. Briefly, orthologous matching requires that the top hit following BLAST all-versus-all searches is the same for multiple data sets, and similarity requires only a unidirectional hit. Download FIG S2, JPG file, 0.2 MB. Copyright © 2017 Zeigler Allen et al. 2017 Zeigler Allen et al. This content is distributed under the terms of the Creative Commons Attribution 4.0 International license.

The ambient viral pool (0.1 μm to 50 kDa) showed a relative increase in the numbers of podoviruses (~46.5% of *Caudovirales*) compared to the cell-sized fraction (~11.0% of *Caudovirales*), largely consisting of those infective for *Pelagibacter* sp., which is among the most abundant bacterial taxa in the Baltic Sea samples; the data are suggestive of an elevated level of host biomass providing a persistent presence of phage regardless of the position within environmental gradients. Podoviruses also represented the dominant cyanophage family in two sites, GS679 and GS695. These sites contrast with regard to salinity and displayed differing cyanobacterial host populations within the *Synechococcus* genus, as determined using phylogenetic placement against a reference tree of concatenated conserved genes (32, 33). On the basis of these data, site GS679 was dominated by brackish strains related to the outgroup in *Synechococcus* phylogenetic studies, *Synechococcus* sp. strain WH5701 (marine subcluster 5.2 [34, 35]), and site GS695 by strains related to *Synechococcus* CC9311 (marine subcluster 5.1B [34, 36]) (Fig. S3; data for all nodes and the associated taxonomy levels achieved are given in Table S2). GS695 and GS679 are the most similar to one another with regard to phage function potential (Fig. S4), which may stem from the presence of cyanophage core genes within these libraries; e.g., viral photosystem genes are well represented for these sites (Fig. S3).

10.1128/mSystems.00125-16.3 FIG S3  (A) Major groups of viruses that contain cyanophage from all virus size fractions. (B) Bacteriophage photosystem genes detected using HMMs that were built from all cyanophage reference sequences. (C) Cyanobacteria relative abundances following phylogenetic placement on the bacterial reference tree. Data were normalized by total bacterial peptides recovered from each site, and taxa that had ≥30 sequences are shown. The taxonomy given is the highest taxonomy (terminal node) achieved during node placement; e.g., *Cyanobacteria*-only sequences were able to clearly be identified by the internal cyanobacterial (phylum) node. Correlations are below 0.05 using the one-sided correlation test (cor.test [R statistics]) for panels B and C. Download FIG S3, JPG file, 0.4 MB. Copyright © 2017 Zeigler Allen et al. 2017 Zeigler Allen et al. This content is distributed under the terms of the Creative Commons Attribution 4.0 International license.

10.1128/mSystems.00125-16.4 FIG S4  KEGG functional categories based on similarity to reference Kegg orthologs (KO). The relative abundance of each category is indicated for sequences from virus-size fraction. Download FIG S4, JPG file, 0.2 MB. Copyright © 2017 Zeigler Allen et al. 2017 Zeigler Allen et al. This content is distributed under the terms of the Creative Commons Attribution 4.0 International license.

10.1128/mSystems.00125-16.9 TABLE S2  Cyanobacteria recovered from RNA-seq data represented as a percentage of the total sequences recovered per site. Download TABLE S2, XLSX file, 0.05 MB. Copyright © 2017 Zeigler Allen et al. 2017 Zeigler Allen et al. This content is distributed under the terms of the Creative Commons Attribution 4.0 International license.

Taxonomic binning of assembled contigs of >5 kb was also used to assess diversity within the virus size class (0.1 μm to 50 kDa; coassembly of all samples). The highest percentage of assembled sequences was classified within the *Siphoviridae* family (35.5%; Fig. S5), unlike what was seen in the read-based analysis, where this taxonomic family represented one of the less populous bacteriophage groups within the *Caudovirales* (4.4%). This could potentially be due to the low diversity within this group contributing to greater assembly success with adequate depth of closely related sequences. The assembly consisted largely of sequences similar to those of nonmarine phages, e.g., *Salmonella*, *Staphylococcus*, and *Streptococcus* phages, and of the marine siphovirus *Cyanophage* PSS2 (37). The next most abundant family in this analysis was *Podoviridae*, and the sequences of the members of that family generally related to those of the *Pelagibacter* phage podovirus (HTVC010P [38]) and *Roseobacter* phage (39, 40).

10.1128/mSystems.00125-16.5 FIG S5  Relative abundances of taxa determined using contigs of >5 kb from virus-size fraction. Taxonomic classification was assigned based on the majority of annotated sequences along the contiguous sequence. Download FIG S5, JPG file, 0.1 MB. Copyright © 2017 Zeigler Allen et al. 2017 Zeigler Allen et al. This content is distributed under the terms of the Creative Commons Attribution 4.0 International license.

### Virus-host inference.

**FIG 2:**
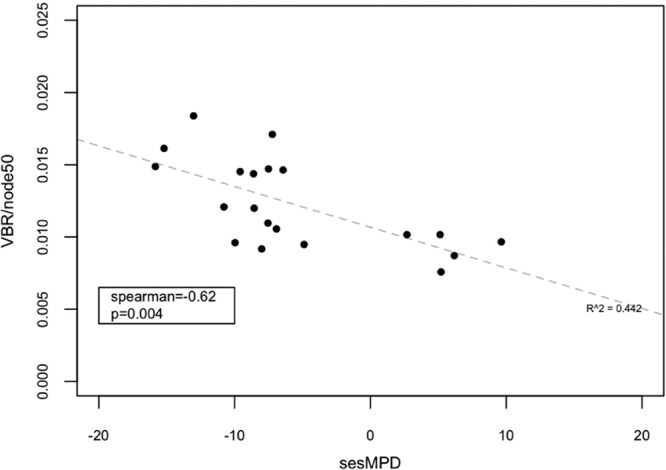
Viral relationship to potential host community structure. The virus-bacterium ratio (VBR) per node50 was used as the metric of infection potential (*y* axis). Normalized mean pairwise distance (sesMPD) values were used for each site as a proxy of host evenness (*x* axis). VBR values were calculated from the relative abundance of viral sequences (bacterial viruses were identified by the first taxonomic level indicative of a potential host identified via APIS) divided by the relative abundance of bacterial sequences (identified via APIS) from the cellular size class (200 to 0.1 µm).

Viruses are thought to play a crucial role in marine aquatic ecosystems by altering microbial community diversity structure, host metabolic status, and cycling of nutrients via host lysis (41–47). We examined viral sequence signatures, i.e., annotated sequences with similarity to known bacteriophage, to address the niche differentiation that may constrain viruses as a result of the use of abiotic (oceanographic contextual data) or biotic (putative host) sources. Sequence data provide a more robust and sensitive assessment of viral identity than the more traditional tools, such as epifluorescence microscopy, which does not account for putative host type and underestimates populations based on size and nucleic acid biases (48). The virus/bacterium ratio has been used in natural and laboratory systems as a metric of the relationship between viral and bacterial populations as well as of infection potential (49–53). In natural communities, however, these phenomena become convoluted due to other prevailing hypotheses. For example, the “dilution effect” hypothesis states that as the number of host species (richness) increases the infection potential decreases; however, there can also be the opposite effect, or “rescue effect,” where high species diversity increases infection potential due in part to more hosts being competent reservoirs for the viruses present (54–58). Using bacteriophage sequence signatures, we sought to evaluate the potential influence of viruses on bacterial community diversity by linking the infection potential (virus-bacterium ratio [VBR] per host richness, calculated as the number of the most abundant nodes that account for 50% of the reads assigned to core genes in each sample [node50]) to bacterial diversity (standardized effect size mean pairwise distance [sesMPD]) (59). In other words, the rationale for infection potential (VBR/node50) is based on two factors: (i) the number of viruses relative to the number of bacteria and (ii) how many hosts—either susceptible or nonsusceptible—are present. For many samples, these estimates were negatively correlated, indicating a possible link between virus infection potential and bacterial diversity (Fig. 2 and Table S3). Increases in VBR/node50 values and decreases in bacterial sesMPD values suggest that virus infection is impacting host diversity. All sites with data indicating a high potential of infection events (VBR/node50) displayed correspondingly low bacterial phylogenetic evenness, suggesting that viruses could be driving mortality of the abundant host lineages present at the time of sampling, reminiscent of the “kill the winner” hypothesis (60). Correlations between sesMPD and VBR/node50 values were negative across all sites (Spearman = −0.62, *P* = 0.004); however, the variability in the data (*R*^2^ = 0.442) is likely due to virus-bacterium interactions accounting for only a portion of the variation in global diversity. Samples with sesMPD values lower than the VBR/node50 values were generally from sites with higher salinity, but no other clear correlations were evident with respect to the environmental data.

10.1128/mSystems.00125-16.10 TABLE S3  Bacterial diversity estimates and estimates of ratios of bacteriophage sequences to bacterium sequences (VBR). Bacterial diversity estimates were derived from phylogenetic placement of core bacterial environmental sequences onto a tree containing all sequenced bacterial representatives. Download TABLE S3, XLSX file, 0.1 MB. Copyright © 2017 Zeigler Allen et al. 2017 Zeigler Allen et al. This content is distributed under the terms of the Creative Commons Attribution 4.0 International license.

**FIG 3:**
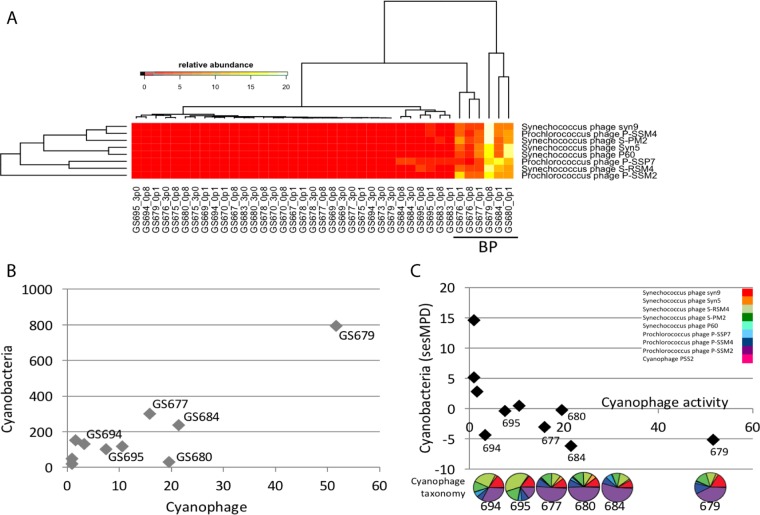
Cyanophage-host interactions from metatranscriptomic data. (A) Major cyanophage populations determined from transcriptomic expression values (relative abundance data represent percentages of reads mapped to ORFs annotated as specific species per site and size class). BP, Baltic Proper. (B) Cyanophage transcript relative abundance (*x* axis; percent cyanophage per site) compared to cyanobacteria numbers found using phylogenetic placement on a bacterial reference tree (*y* axis; predicted number of cyanobacteria present in sample). (C) Cyanophage transcript relative abundance (*x* axis) and *Cyanobacteria* diversity estimates (standardized effect size mean pairwise distance [sesMPD]) derived from phylogenetic tree placement. Taxonomic assignment of cyanophage transcript sequences using similarity is shown in pie charts, and the legend is given in the upper right quadrant. Pie charts are shown for sites with low sesMPD and correspond to those in panel B.

Periodic surface blooms of nitrogen-fixing cyanobacteria (mainly *Nodularia spumigena*, *Aphanizomenon* sp., and *Dolichospermum* sp.) are common in the central and south Baltic Sea (mainly in Baltic Proper) during summer (61–63). In addition to negative effects from harmful toxin production, such blooms in the Baltic Sea are strongly linked to development of widespread benthic hypoxia, which is a major ecosystem health hazard (64). Viral infection of these blooms is therefore of increased interest as this would facilitate biomass recycling in the pelagic zone through the microbial food web via the viral shunt. Phage capable of infecting cyanobacteria were identified and correlated to putative shifts in host community structure using metagenomics and transcriptional activity estimates to assess the microbe-virus interactivity of this prevalent and important group. While host and viral populations were detected throughout the Baltic Sea using metagenomics, viral activity (transcript abundance) was primarily detected within the Baltic Proper (BP) (Fig. 3A). To evaluate viral activity, metatranscriptomic libraries targeting mRNA were constructed using rRNA subtraction and total mRNA amplification, thereby avoiding bias toward transcripts with poly(A) tails. Cyanophage have dsDNA genomes and undergo an mRNA intermediate step during infection; therefore, this method provides an important insight into the populations which are transcriptionally active at the time of sampling. The limited spatial activity of cyanophages (samples GS679, GS680, and GS684) could reflect favorable environmental conditions during sampling of these sites or could indicate an abundant standing stock of cyanophages which are not active until advantageous abiotic or biotic conditions develop. Indeed, samples GS679 and GS680 were taken at a site where a decaying *Nodularia* bloom was observed at the time of sampling. *Prochlorococcus* phage is likely not present in the Baltic Sea at appreciable levels due to the lack of host species seen in previous amplicon studies of the Baltic Sea (19, 20, 65, 66). Our previous study was able to taxonomically assign sequences to *Prochlorococcus* spp.; however, these were largely phylogenetically nonreliable and most are likely more similar to those of other unsequenced Baltic Sea picocyanobacteria within the *Synechococcus* and *Cyanobium* lineages. Further, on the basis of observations during sampling of a *Nodularia* bloom demise, we hypothesize that these sequences belong to *Nodularia* phage, previously identified within the Baltic Sea using the major capsid protein gene as a molecular marker (15), or to another picocyanobacterium phage. Although not within the scope of this study, population-level growth rate measurements could be an important parameter for further understanding virus-host dynamics. Previous reports indicate that fast-growing heterotrophic bacteria occur in lower numbers as a strategy to escape viral predation and are negatively correlated with viral abundance (67). In contrast, slow-growing groups, such as cyanobacteria, are positively correlated with viral abundance. Here, we leveraged metatranscriptomic data to assess interactions using cyanophage transcriptional activity compared to host transcriptional activity and diversity. In general, host number and virus transcript abundance were positively correlated (Fig. 3B, *P* = 0.04, Spearman = 0.59). Further, viral transcript abundance, a proxy for infection activity, and host diversity were negatively correlated in most sites (Fig. 3C, *P* = 0.005, Spearman = −0.79), suggesting a role for viral activity in driving the decline of cyanobacterial host diversity, as shown using (VBR/node50)/sesMPD analyses of whole bacterial/phage communities. These data regarding parasitic pressures on prevalent and important primary producers aid in understanding cyanobacterial bloom dynamics and reoccurrence of microorganisms capable of episodic pulses of toxin production.

**FIG 4:**
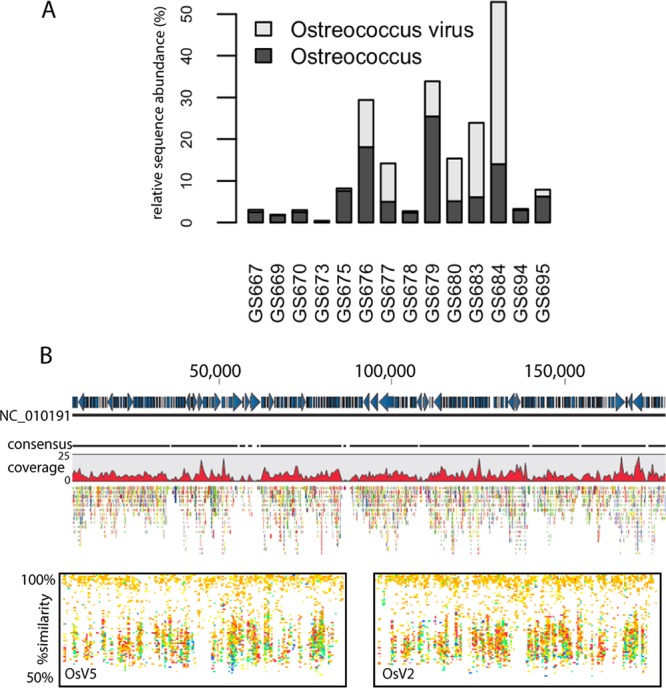
*Ostreococcus* and virus-host interactions. (A) Transcript abundance for sequences binned as *Ostreococcus* (dark gray) and *Ostreococcus* virus (light gray) based on homology at the genus and group levels, respectively. (B) Site, read, and environmental data from reference recruitment determined using *Ostreococcus* virus *OsV5* and *OsV2*. (Top) Recruitment plot of sequences from GS680, 683, and 684, where the majority of sequences were recovered, against sequences of *Ostreococcus* virus *OsV5* (CLC Genomics Workbench). (Bottom) Recruitments from all sites and size fractions for the genomes of *Ostreococcus* viruses *OsV5* and *OsV2*. The dashed line denotes 89% coverage (see reference 92 for coverage cutoff details).

The widely distributed picoeukaryote *Ostreococcus* and its viruses were detected at many sites, with a negative association (high virus sequence abundances relative to host sequence abundances within the data set) at sites GS680 (BP), GS683, and GS684 (Belt Sea, grouped with BW in this study; Fig. 4A). In general, *Ostreococcus* virus *OsV5* was well represented throughout the data set using fragment recruitment analysis and was present at a level of 8× coverage in samples originating from sites GS683 and GS684 and at 18× across all 21 sites (Fig. 4B; for a color scheme of read mappings arranged according to site and salinity, see Fig. S7). *Ostreococcus* virus transcriptional activity was also highest at sites GS683 and GS684 relative to other sites (17.8 and 38.9% of *Ostreococcus* virus transcripts, respectively) and to the transcriptional activity of their hosts (6.0 and 14.0% of *Ostreococcus* transcripts, respectively). The negative correlation of host and virus transcript sequences points to a possible infection event that was captured during sampling at this site. Recently, unique *Ostreococcus* operational taxonomic units (OTUs) were identified within the Baltic Sea, primarily arising in Bothian Bay and Akorna Basin samples, representing unique clades (clades D and C, respectively) (65). Clade D contains the marine species *Ostreococcus tauri*, and our data would suggest that this is the putative host, since viruses *OsV5* and *O. tauri* virus 1 and 2 are the dominant taxa based on homology and their location is within the areas with the higher salinity levels.

### Global virome from transcriptome sequencing (RNA-seq) methods.

**FIG 5:**
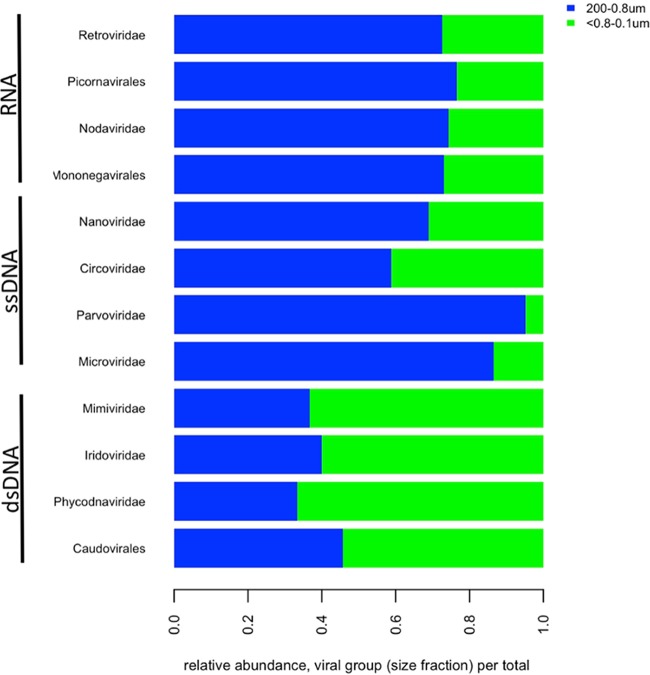
Relative abundance of major viral groups from RNA-seq libraries. Abundances were calculated as viral group per total viral sequences in all groups. Green represents the sequences from the small cellular fraction (<0.8 to 0.1 µm), and blue denotes the collapsed bins from larger-size fractions (200 to 0.8 µm).

Examining viral populations using measures of activity provided insight into putatively active virus-host interactions. This is particularly useful for DNA viruses that are transcribed into an RNA intermediate. However, viruses with RNA genomes may not go through an mRNA intermediate that can be explicitly detected using this method; therefore, we focused our analyses of RNA viruses on distribution and potential associations with hosts rather than transcriptional activity. Throughout the Baltic Sea, viral transcripts were detected for some of the major dsDNA virus-host systems identified using metagenomics and led to the identification of sequences for RNA and single-stranded DNA (ssDNA) not seen previously using traditional metagenomic techniques, thus providing a new view of the global Baltic Sea virome. Of note, ssDNA and RNA viruses have much smaller genomes (midpoint among genomes of ca. 6 and 17 kb, respectively) than dsDNA viruses (midpoint ca. 600 kb); therefore, lower relative sequence abundances may be due to genome size rather than ecological significance. Overall, numbers of viral sequences from transcriptomes ranged from approximately ~140,000 to 1.9 million reads per site and size class (8.6 million total viral sequences; Table S1C), for an overall total of 3.2% viral sequences per total sequences recovered. In most sequence libraries, dsDNA viruses were the dominant genome type, comprising primarily *Caudovirales* (bacteriophage) and *Phycodnaviridae* (large eukaryotic phytoplankton virus) groups (Fig. S6). The higher relative abundance of ssDNA and ssRNA viral sequences detected using the 3.0- and 0.8-μm-pore-size filters suggests that their putative hosts are of larger cell size and are likely microeukaryotic in origin (Fig. 5). Recent reports from cultivation studies (68–71) and cultivation-independent studies (72, 73) suggest that microeukaryotic hosts may be the primary microbial prey for marine RNA viruses. Conversely, dsDNA viral sequences were more prevalent within the 0.1-to-0.8-μm size class and most likely originated from either bacteriophages or picoeukaryotic phytoplankton viruses that were retained due to increased particle size (Fig. 5 and Fig. S6A).

10.1128/mSystems.00125-16.6 FIG S6  Taxonomic bins from RNA-seq data. (A) Major groups across site and size classes from each filter (using pore sizes of 0.1, 0.8, and 3.0 µm). (B) Total transcript taxonomic relative abundances. (C) Reference maximum likelihood phylogenetic tree of RNA-dependent RNA polymerase (RdRP) gene with environmental sequenced phylogenetically placed analysis (pplacer; see Matsen et al. [91]). Environmental sequence bins (circles) mapping to the marine eukaryotic algal reference clade are shown in green. Download FIG S6, JPG file, 0.5 MB. Copyright © 2017 Zeigler Allen et al. 2017 Zeigler Allen et al. This content is distributed under the terms of the Creative Commons Attribution 4.0 International license.

10.1128/mSystems.00125-16.7 FIG S7  Color scheme for read colors in Fig. 4 genome recruitment plots. Download FIG S7, PDF file, 0.02 MB. Copyright © 2017 Zeigler Allen et al. 2017 Zeigler Allen et al. This content is distributed under the terms of the Creative Commons Attribution 4.0 International license.

*Microvirus* and *Circovirus* strains have ssDNA genomes and have been discovered in diverse marine environments and hosts (74–76), although they are typically selected through the bias associated with sequence amplification strategies, particularly using phi29 polymerase and whole-genome amplification as used during sequence library preparation (77, 78). Here, RNAseq provided an unbiased assessment of ssDNA virus transcript activity. While most sites had low (0 to 9%) abundance values, one sample from a site at a freshwater lake, Lake Torneträsk (GS667), showed a substantial increase in recovered microvirus sequences (Fig. 5 and Fig. S6), representing a diverse group of bacteriophage, primarily associated with the 3.0-to-0.8-μm size class, and accounted for 43.7% of all ssDNA sequences recovered. Bacteriophage have been identified within larger size classes (9) and are proposed to be associated with particle-associated bacteria (32); therefore, our data suggest that the *Microviridae*-related sequences obtained here are likely associated with bacteria that were retained on the 3.0- and 0.8-μm-pore-size filters. The members of the *Parvoviridae* represent an additional ssDNA group with wide distribution in the Baltic Sea, with particular enrichment in the northern Baltic Sea (BB and BS) and in the 74-m-deep sample within the BP (GS678, Landsort Deep) (Fig. 5). The members of this family infect both invertebrate and vertebrate hosts, including fish, tetrapods, and insects. In these samples, almost all sequences were binned into *Porcine parvovirus*, which causes reproductive failure in swine populations. Industrial pig farms are numerous along coastlines of the southern Baltic Sea, which functions as a drainage area for runoff, including possible associated microbes, viruses, and nutrients (79).

Both dsRNA and ssRNA viruses were present at various sequence abundances within all of the samples. Of note, these sequences may originate from individual genomes rather than from transcripts. Generally, the greatest taxonomic representation was that of dsRNA-like sequences and marine algal viruses, using phylogenetic placement on an RNA-dependent RNA polymerase (RdRP) reference tree (Fig. S6C; green). Additionally, sequence similarity revealed a high proportion of *Retroviridae*-like sequences within the RNA viral groups; however, they comprised only 0.6% of total viral transcripts. The members of this family consist of diverse viruses, including certain fish viruses. Additionally, *Picornavirales* species were detected within the marine BW and brackish BP waters, whereas members of the *Mononegavirales* and *Ourmiavirus* groups were detected further northward. These groups had not been detected previously, in any appreciable amount, using sequence-based similarity approaches in GOS omics-derived data.

### Viruses as predictors of ecosystem health.

One of the most powerful applications of DNA and RNA sequencing for Baltic Sea studies is related to potential assessments of ecosystem health. The use of omics-related data to develop indices of ecosystem health holds great promise. Once suitable baselines are established, such methodology could be employed for rapid and relatively inexpensive determination of perturbations. Within our data set, a relatively high portion of transcripts of fish viruses were detected within the range between 0.01 and 4.4% of total transcripts (the range of all pathogen averages detected was 0.05 to 1.7%). These viral sequences were similar to those of members of diverse families and host ranges, such as *Nodoviridae* and *Retroviridae* (RNA) and *Iridoviridae* (DNA) (Table S1C), which have the potential to cause devastation to local fisheries.

**FIG 6:**
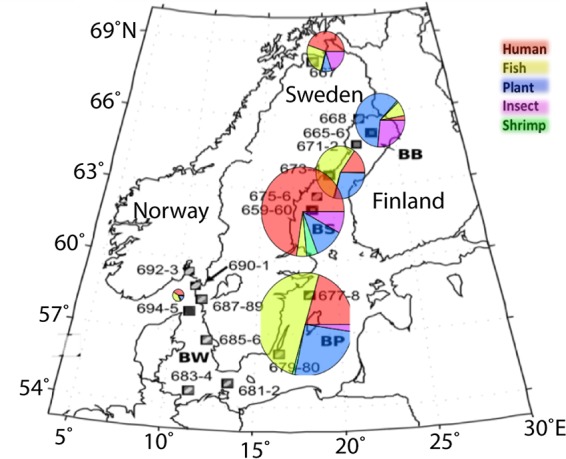
Baltic Sea pathogenic viruses. These were identified using homology searches against an in-house database, PhyloDB, that comprises all sequenced genomes as well as all available eukaryotic transcriptomes. Viral sequences were grouped based on infectivity of similar hosts. The size of each pie chart is proportional to the total number of putative viral pathogenic sequences from the region.

Human viral pathogens, many capable of causing gastroenteritis, specifically, *Picobirnavirus* and *Norovirus*, were also detected using sequence-based similarity (Fig. 6). In addition, *Giardia lamblia* virus and hepatitis E virus, causing gastrointestinal pain and liver disease, respectively, were identified particularly within BP and the BW. All of these pathogens are known to be commonly transmitted via contaminated drinking water. Human coronavirus and rhinovirus A, capable of causing respiratory illness and the common cold, were also found among sites within BP and BW. Human influences are also detected, i.e., the swine viruses from agriculture practices potentially polluting the northern sites and concentrating the pollution in the Baltic Proper. These data provide a first glimpse at the use of meta-omics for the study of human impacted regions, and more statistically robust phylogenetic and epidemiology data will be required to truly understand the role that these pathogens play in relation to the natural biodiversity and human threats.

## DISCUSSION

Coupled metagenomic (potential) and metatranscriptomic (transcriptional activity) analyses of dominant dsDNA viral populations enabled a unique look at the distribution of active viruses in the wider Baltic Sea area. Cyanophage sequences are found at appreciable levels in most surface and photic zone samples; however, transcriptional activity was primarily limited to central Baltic Proper. This approach also allowed us to identify sites of increased activity of major environmentally relevant taxa, *Cyanobacteria* and the picoalga *Ostreococcus*, as well as of viruses, suggesting a way to facilitate future detailed studies of gene transfer and interplay between these groups. Using sequence-based metrics of infection potential (VBR/node50) and host richness (sesMPD), we were able to circumvent issues associated with traditional microscopy counts, e.g., the inability to discern taxonomic affiliation and loss of viruses with small genomes due to diminished fluorescence. However, our approach is dependent on robust database support for taxonomic assignments and therefore could potentially underestimate viral abundance. Notably, samples analyzed in this study were dominated by marine bacteriophage that are well represented in current databases, e.g., *Pelagibacter* phage and cyanophage; however, we do recognize that this is a possible limitation of the analysis. Furthermore, increased time-series efforts will shed new light on the dynamic virus-host systems that shape the Baltic Proper through their yearly reoccurring cyanobacterial bloom and bust cycles.

In addition to the dsDNA virome, our methodology facilitated recovery of other more enigmatic RNA virus populations that have remained elusive due in part to size, cultivability, and achieved sequence depth limitations. RNA viruses were detected within the Baltic Sea metatranscriptomic sequence libraries at an appreciable level. These data could pave the way for new biomarkers which are necessary for establishment of fundamental baselines concerning marine RNA virus diversity and distribution. Such approaches will ultimately provide a greater understanding of the human influence on the natural biodiversity of the Baltic Sea ecosystem. Similarly, this study provided an unbiased (compared to previous reports) assessment of transcriptionally active ssDNA viruses present within the Baltic Sea samples and highlighted the relative overabundance within certain sites, such as Lake Torneträsk (GS667) and the Landsort Deep (GS678).

Viruses are known to modulate community structure through infection cycles, which leads to various chemical fluxes within the system resulting in transformed biogeochemical cycles. Sequence data provide evidence that can be extrapolated to gain a greater understanding of the viral ecological niche. Using the VBR, normalized by the number of abundant nodes (taxa) on a bacterial core gene phylogenetic tree, as a metric of infection potential, we found that this potential was most often negatively correlated with the Baltic Sea bacterial diversity. This supports the prediction that greater host diversity results from a negative-density-dependent selective environment. Consequently, more-abundant and more-efficient host populations may have an increased susceptibility to viral attack. Therefore, this scenario may lead to a direct relationship between host diversity and risk of infection. The dilution effect hypothesis is well supported in human infection disease models, where greater host diversity leads to a reduced risk of infection (55, 80). Other evidence suggests that biodiversity loss in a system may increase disease or, conversely, that high biodiversity may serve as a pathogen source (81). These data support the idea that maintenance of biodiversity is crucial for promoting ecosystem health, in particular, within the Baltic Sea, where the influx of anthropogenic viral sources may lead to the loss of species and ultimately to unbalanced feedback. Together, the increases in exogenous microbial and viral populations may lead to increased rates of disease and evolution that surpass key ecological thresholds, pushing the ecosystem to its tipping point. This study showed the potential for improved use of omics-derived data as part of risk assessment and management of marine ecosystems, including the Baltic Sea.

## MATERIALS AND METHODS

### Sample acquisition.

Samples were obtained during a 2009 expedition of Sorcerer II detailed by Dupont et al. (19). Briefly, 200 liters of seawater was collected, prefiltered (using a 200-μm-pore-size Nytex net), and serially filtered using 3.0-, 0.8-, and 0.1-μm-pore-size impact filters. Virus-sized samples were obtained through concentration of the water produced from 0.1 μm-pore-size filters via tangential flow filtration (Pellicon Maxi Cassette; Millipore) (50 kDa). Filters were stored in DNA extraction buffer with RNAlater (Life Technologies, Inc.). Viral concentrates (VCs) had 20% molecular grade glycerol added. All samples were frozen onboard and transferred on dry ice.

### DNA isolation and preparation of sequence libraries.

For detailed methods describing DNA extraction from GOS-derived filters, see reference 92. For details of the preparation of viral DNA from VCs for sequencing using 454 GS FLX titanium sequencing platform, see the methods described by Williamson et al. (9). Briefly, environmental genomic DNA was sheared using a Covaris instrument and size selected to 500 to 800 bp. Linkers were ligated to DNA fragments, and amplification was performed using 15 cycles in triplicate. Sequencing adaptors were ligated to the amplified products and purified using AMPure beads prior to sequencing using a 454 GS FLX titanium sequencing platform. Viral metagenomic sequences were trimmed for both the linker and adaptor. Artificial replicates were screened for and removed using the approach described by Gomez-Alvarez et al. (93). To screen for cellular contamination, viral particles were subjected to DNase treatment (3×) and RNase treatment (1×) followed by PCR using universal primers for 16S ribosomal DNA (rDNA). Once sequencing was complete, metagenomic data were screened to identify genome equivalents using hidden Markov model (HMM) searches of conserved genes (most of which were single-copy genes; see reference 32 for more information on bacterial genome equivalents).

### Metagenomic viral library assembly.

Sequences from virus-size samples (0.1 μm to 50 kDa) derived from 454 metagenomic sequence libraries were assembled together using a Newbler assembler (Roche). Prior to assembly, sequences of low complexity were identified and removed using DUST (82). The sequences were first assembled using Newbler with default parameters, including the minimum identity (–mi) set at 86 and the –rip option, which outputs each read into only one contig. Following the initial assembly, downsampling or bioinformatics normalization was performed as previously described by Allen et al. (83). Briefly, areas of the genome with high coverage were randomly reduced to within 2 standard deviations (SDs) of the average contig coverage. These methods were implemented to increase assembly metrics, i.e., fewer contigs, greater length, and greater N50 scores.

### Metagenomic sequence annotation.

Sequencing reads from the filters were processed as previously described. Virus-size fractions were first processed through FragGeneScan (84) for open reading frame (ORF) calling using the 454-10 train file, given an approximate error rate of 1%. Taxonomic profiles were generated using the Automated Phylogenetic Inference System (APIS), which generates phylogenetic trees from top BLASTp hits (E value, ≤1e−9) from PhyloDB. Subsequently, the closest relative becomes the taxonomic lineage for the query (environmental) sequence annotation. The use of APIS for metagenomic sequences has been previously described (9, 85). Additional functional information was derived from HMM, KEGG, and GOS cluster searches. Photosystem genes were identified using BLAST (E value, ≤1e−3) against a boutique database consisting of cyanophage reference sequences from PhyloDB for *psaA*, *psaB*, *psaC*, *psaD*, *psaE*, *psaE*, *psaF*, *psaJ*, *psaK*, *psbA*, and *psbD*.

### PhyloDB.

This custom database is comprised of peptides obtained from KEGG, GenBank, JGI, Ensembl, CAMERA, and various other repositories. Altogether, version 1.076 of the database consists of 29 M nonredundant proteins from 139 k viral, 15 M bacterial, 446 k archaeal, and 13 M eukaryote species, including 9.8 M from the Gordon and Betty Moore Foundation-funded Marine Microbial Eukaryote Transcriptome Sequencing Project (MMETSP; http://marinemicroeukaryotes.org/). PhyloDB can be downloaded at the following URL: https://scripps.ucsd.edu/labs/aallen/data/ (see “Databases and Collections”).

### RNA isolation and preparation of sequence libraries.

RNA samples (200 liters) were collected onto 293-mm-diameter Supor filters with serial filtration using 3.0-, then 0.8-, and then 0.2-μm-pore sizes. RNA was purified using Trizol reagent (Life Technologies, Inc., Carlsbad, CA), treated with DNase (Qiagen, Valencia, CA), and cleaned with an RNeasy MinElute kit (Qiagen, Valencia, CA). RNA quality was analyzed with a 2100 Bioanalyzer and Agilent RNA 6000 Nano kits (Agilent Technologies, Santa Clara, CA) and quantified using a Qubit fluorometric quantification system (Thermo Fisher, Waltham, MA).

Total mRNA transcriptome libraries were made with a ScriptSeq v 2 RNA-Seq kit (Illumina Inc., San Diego, CA) using subtractive hybridization of rRNAs with antisense rRNA probes (94) recovered via PCR using 0.1-, 0.8-, and 3.0-µM-pore-size filters and a mixture of DNA obtained from the thirteen Baltic sampling stations comprising this study. A 250-ng volume of total community RNA was used for subtractive hybridization. Multiple rounds of subtractive hybridization of rRNAs were used to obtain at least 30 ng of rRNA-depleted total RNA. The quality of the rRNA-depleted total RNA was analyzed on a 2100 Bioanalyzer with Agilent RNA 6000 Pico kits (Agilent Technologies, Santa Clara, CA). A 5-ng volume of rRNA-depleted total RNA was used as an input for a SciptSeq v 2 RNA-Seq kit (Illumina Inc., San Diego, CA) following the protocol of the manufacturer. A total of 10^7^ copies of ArrayControl RNA spikes (Life Technologies, Inc., Carlsbad, CA) were added to each sample prior to ScriptSeq amplification. AMPure XP beads (Beckman Coulter, Inc.) were used for cDNA and final library purification. Library quality was analyzed on a 2100 Bioanalyzer with Agilent high-sensitivity DNA kits (Agilent Technologies, Santa Clara, CA). The resulting libraries were subjected to paired-end sequencing via the use of an Illumina HiSeq system.

For poly(A) mRNA transcriptomes, 0.8 μg of total community RNA and 2 × 10^9^ copies of ArrayControl RNA spikes (Life Technologies, Inc., Carlsbad, CA) were amplified using TruSeq RNA library preparation kit v 2 (Illumina), following the protocol of the manufacturer with minor adjustments. Specifically, the fragmentation time was modified according to RNA quality. Library quality was analyzed on a 2100 Bioanalyzer with Agilent high-sensitivity DNA kits (Agilent Technologies, Santa Clara, CA). The mean size of the libraries was around 400 bp. The resulting libraries were subjected to paired-end sequencing via the use of an Illumina HiSeq system.

### Transcriptome assembly.

Reads were trimmed for quality and filtered to remove primers, adaptors, and rRNA sequences using Ribopicker v.0.4.3 (86). *De novo* assembly of Illumina HiSeq reads into contigs was accomplished with multiple rounds of assembly using CLC Assembly Cell (CLC bio) clc_novo_assemble. Samples were individually assembled, and ORFs were predicted on contigs using FragGeneScan (84). Read counts for each ORF per each sample were obtained by mapping reads to predicted ORFs using CLC Assembly Cell (CLC bio) clc_ref_assemble_long.

### Transcriptome annotation.

ORFs were annotated *de novo* for function via KEGG, KO, KOG, Pfam, and TIGRfam assignments. Taxonomic classification was assigned to each ORF using the reference data set PhyloDB (see “PhyloDB” above). Sequences from both the poly(A)- and random hexamer-derived libraries were pooled at the annotation step for site and size class. Further, pathogen annotation was conducted using literature searches of the best species/strain hit for evidence of infectivity of fish, shrimp, human, plant, or insect species.

### Virus-to-bacterium ratio and host diversity.

Annotated metagenomic sequences were taxonomically binned into groups of viruses that infect bacteria based on similarity to known bacteriophage and bacteria using APIS. node50 is a measure of taxonomic richness derived from the distribution of core genes in a sample assigned to nodes in a reference tree and is calculated as the number of the most abundant nodes that account for 50% of the reads assigned to core genes in each sample. The mean pairwise distance (MPD) was calculated from a phylogenetic placement 16S rDNA tree (see reference 87 for methods), where MPD is the tree distance between all pairs of hits within a sample. To account for sample size, a normalized MPD (the standardized effect size MPD [sesMPD]) metric was used for determinations of bacterial diversity (88–90). The sesMPD value represents the number of standard deviations (SDs) by which a value for a sample deviates from the average for samples of the same size taken from the entire fraction. The calculation for determining sesMPD is as follows: sesMPD = [(observed MPD) − (mean randomized MPD)]/(SD of randomized MPD). One limitation of the MPD metric is that some samples have many more hits within the bacterial tree and, thus, more pairs; therefore, MPD can be skewed by sample size. The sesMPD is used to attempt to normalize these numbers by comparing the MPD of a sample with the average MPD from randomized subsampling (100× for these data) of the same size taken from all the samples mixed together. All correlation coefficients were calculated using cor.test (R; http://www.R-project.org) using the Spearman method and one-sided *P* values. For cyanobacteria/cyanophage comparisons (Fig. 3), bootstrap analysis (1000 iterations) was performed to confirm that the positive and negative correlations were within the 90% confidence interval.

### Accession number(s).

The metatranscriptomic data were deposited at NCBI GenBank SRA under BioProject accession no. PRJNA320636. The metagenomic libraries were deposited in iMicrobe under project code CAM_P_0001109.
