# Supplementary figures and images for: The Baltic Sea Virome: Diversity and Transcriptional Activity of DNA and RNA Viruses
Source: mSystems. 2017 Feb 14;2(1):e00125-16. doi: 10.1128/mSystems.00125-16 (PMC5309335; doi:10.1128/mSystems.00125-16)

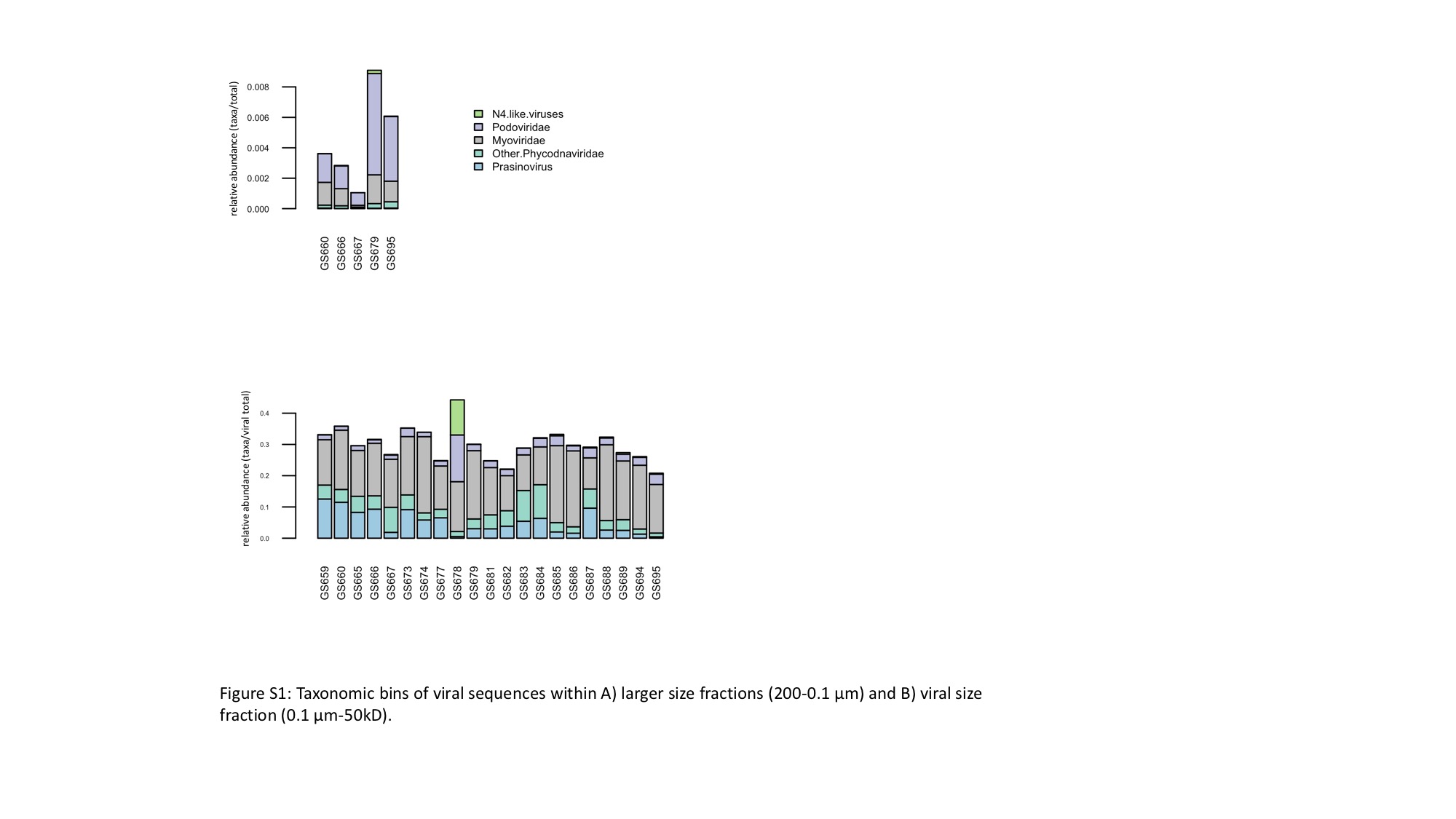

Supplement: FIG S1 [file sys001172085sf1.jpg]

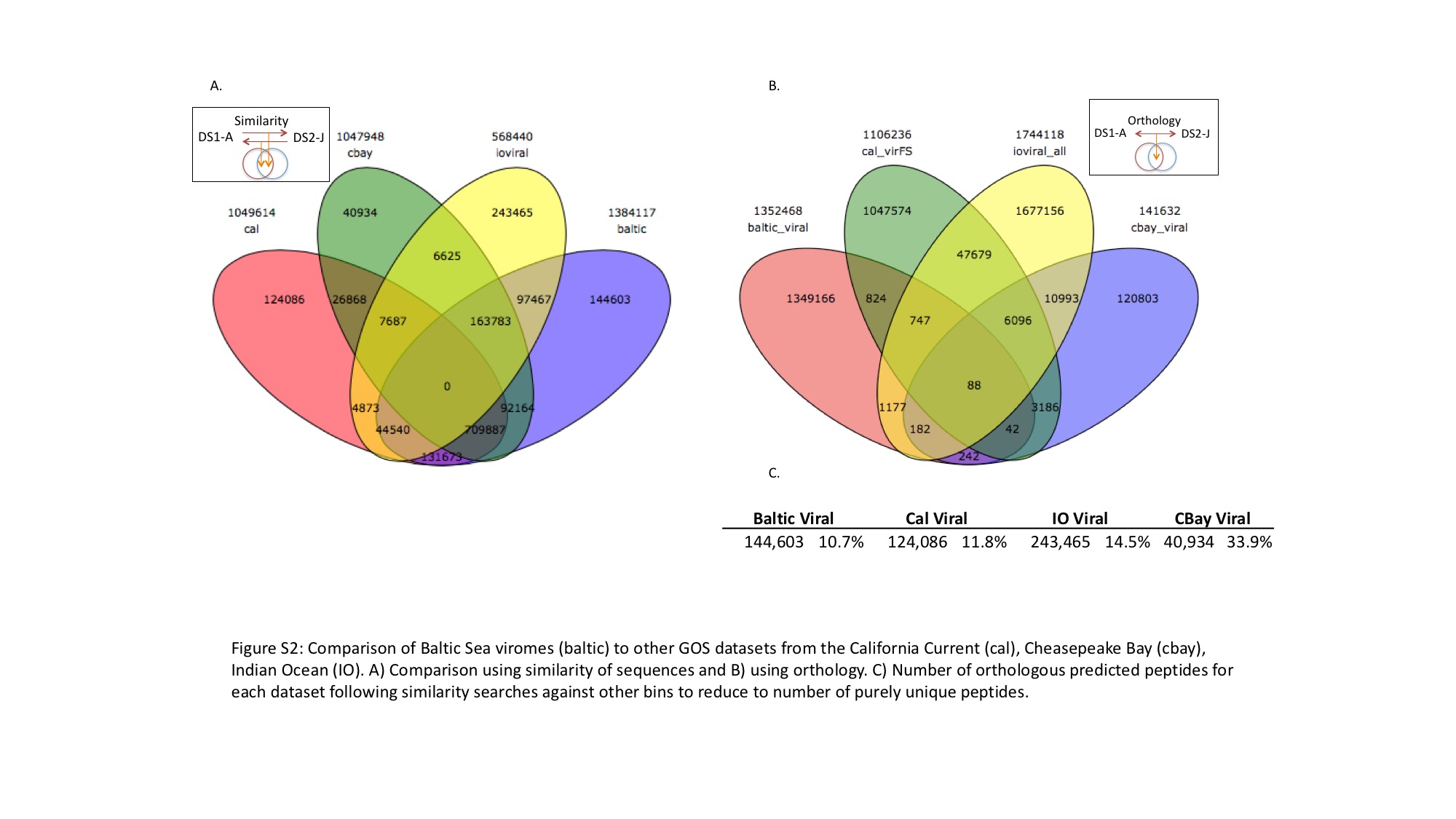

Supplement: FIG S2 [file sys001172085sf2.jpg]

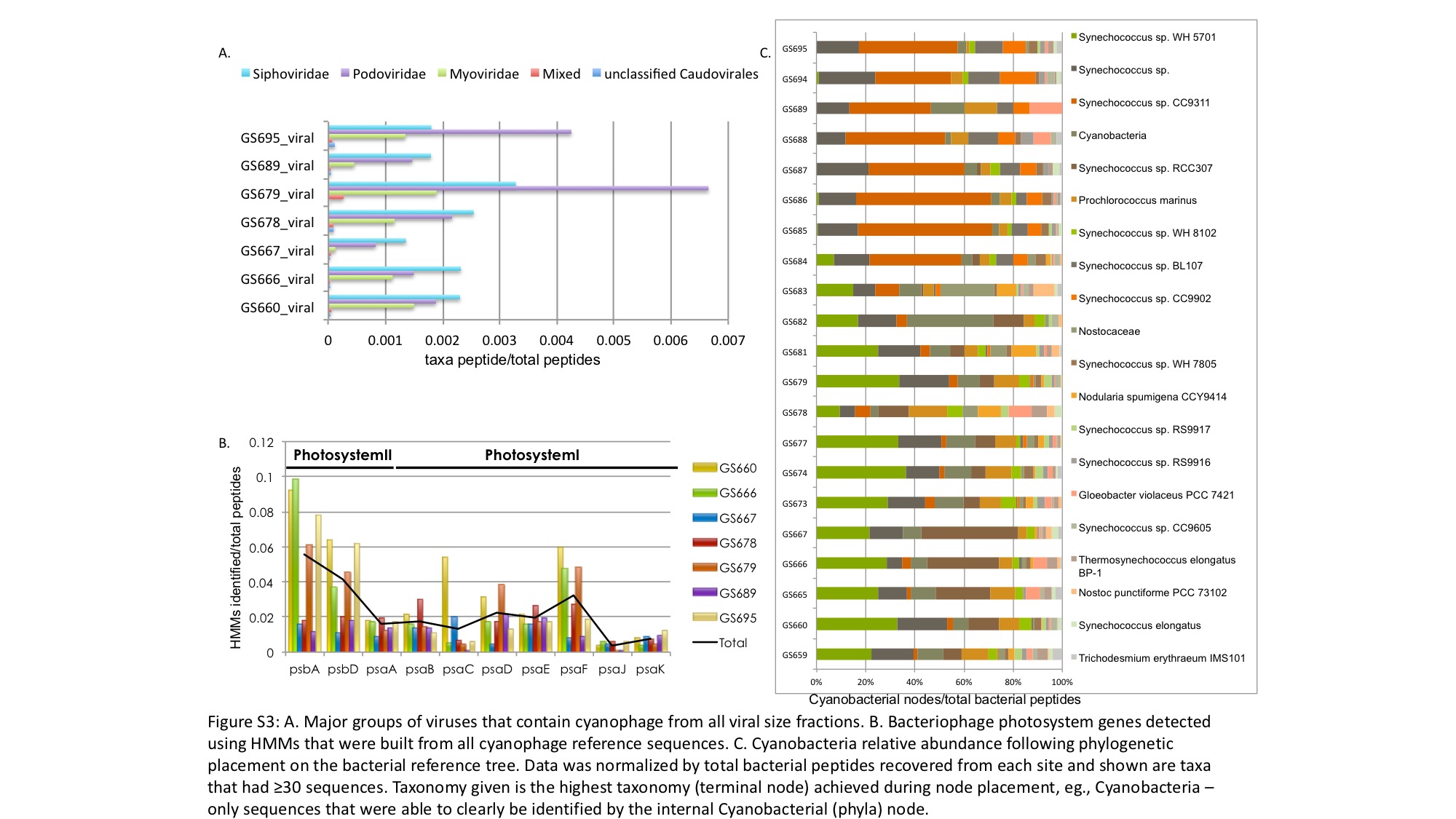

Supplement: FIG S3 [file sys001172085sf3.jpg]

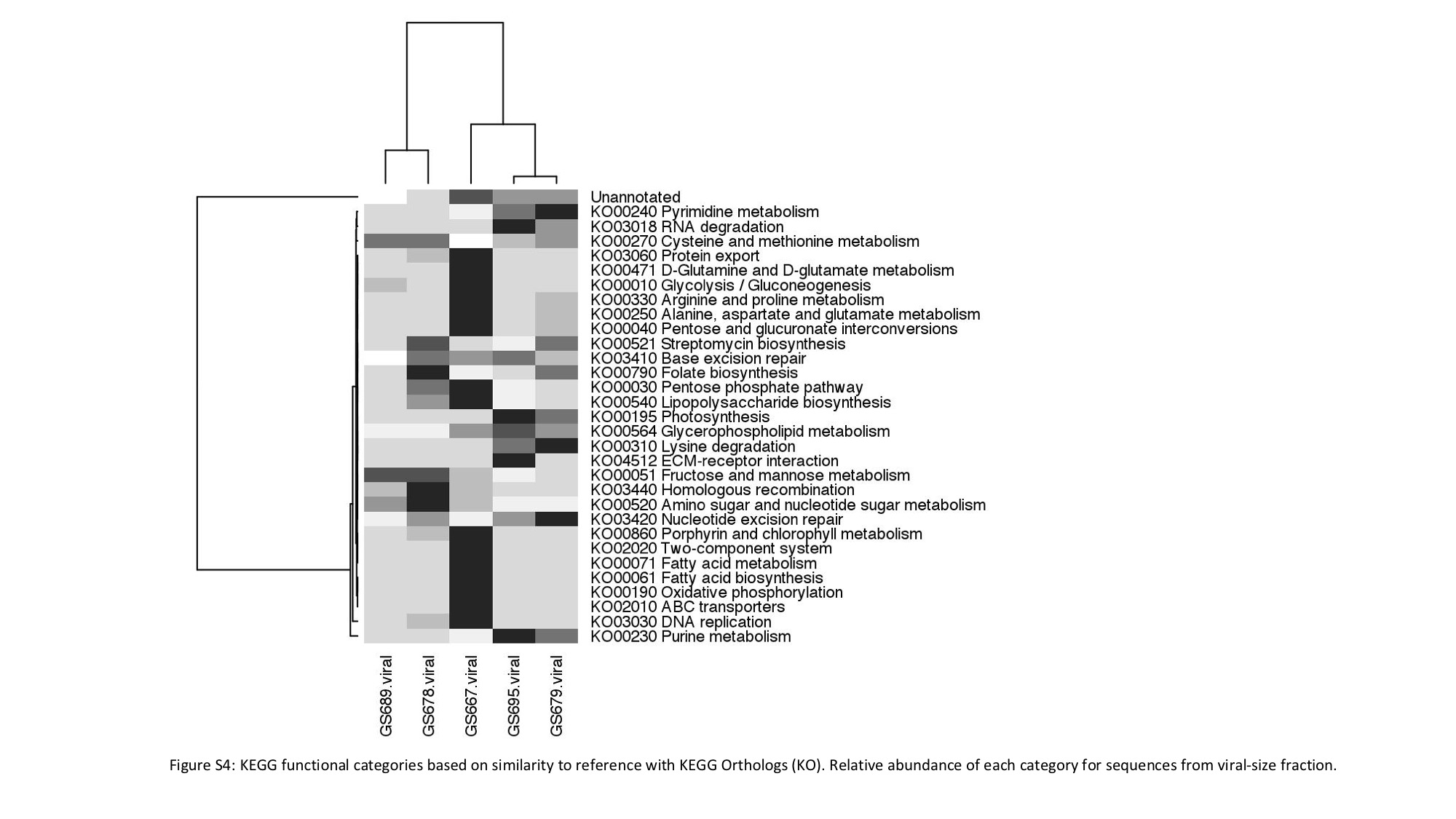

Supplement: FIG S4 [file sys001172085sf4.jpg]

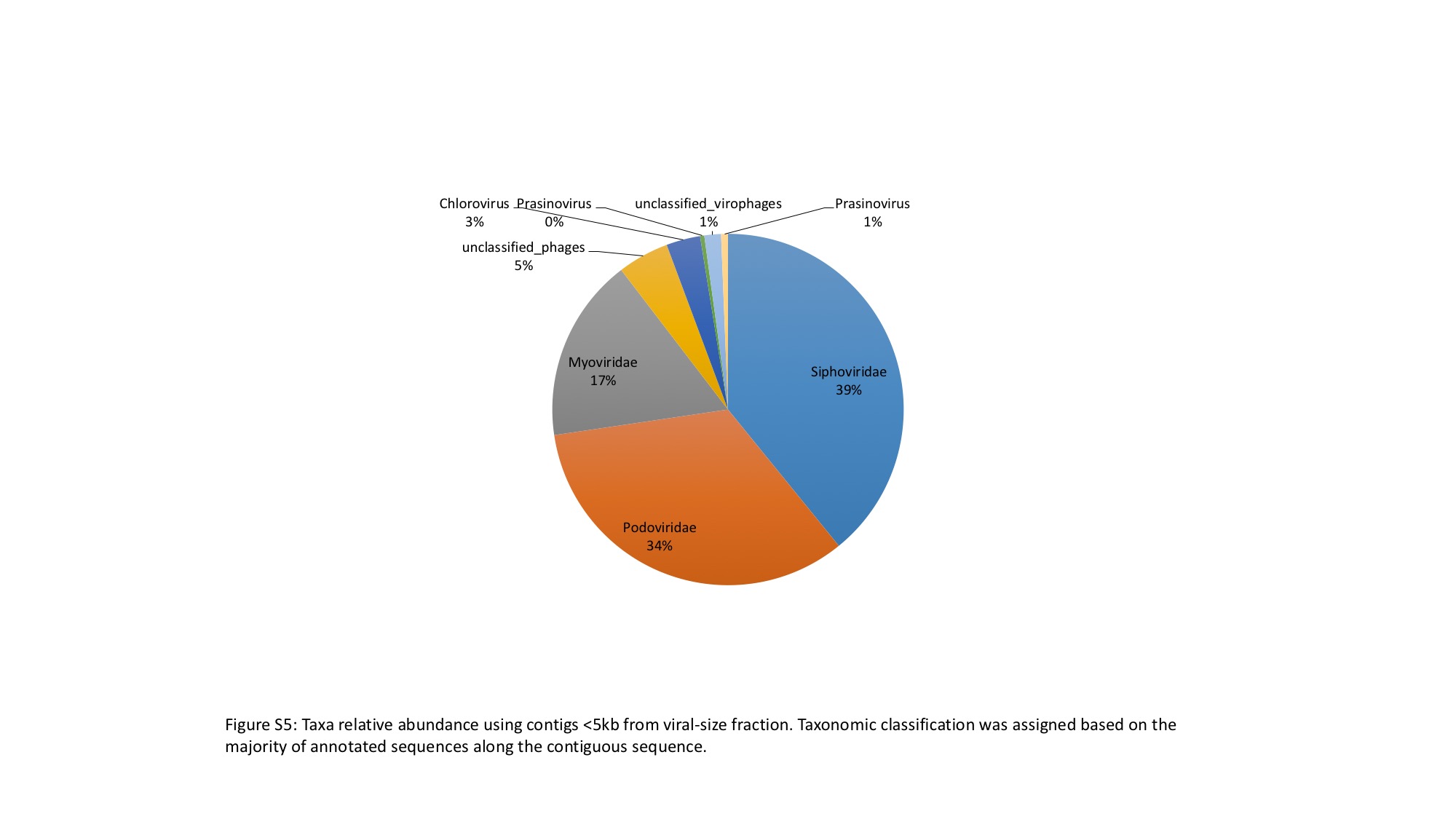

Supplement: FIG S5 [file sys001172085sf5.jpg]

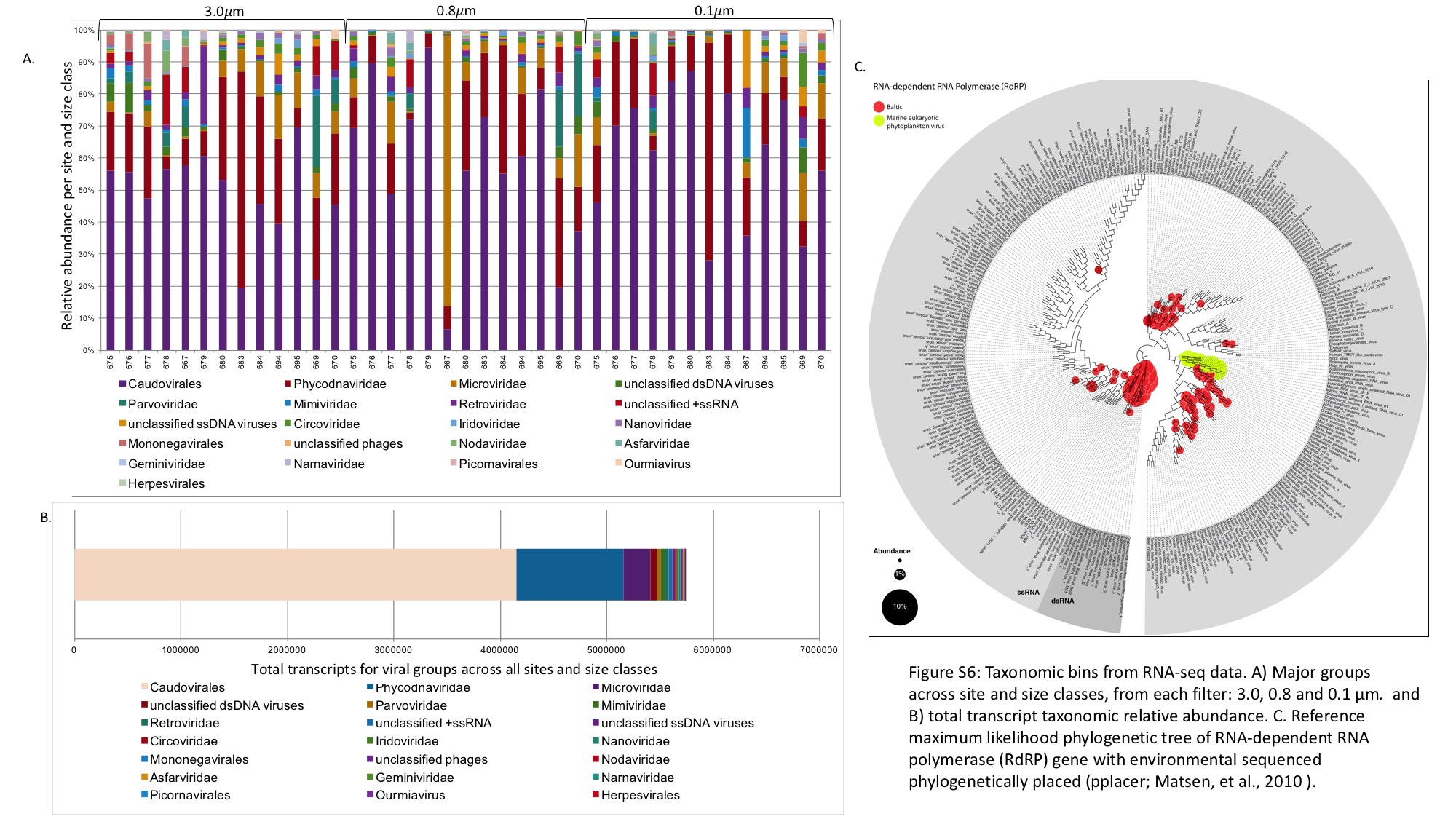

Supplement: FIG S6 [file sys001172085sf6.jpg]
