# Supplementary material for: The Baltic Sea Virome: Diversity and Transcriptional Activity of DNA and RNA Viruses
Source: mSystems. 2017 Feb 14;2(1):e00125-16. doi: 10.1128/mSystems.00125-16 (PMC5309335; doi:10.1128/mSystems.00125-16)
Supplement: FIG S7 [file sys001172085sf7.pdf]

GS667_G9JOFJ3_3p0
GS667_G9JOFJ3_0p8
GS667_GCVIDIU02_0p1
GS667_GQY5VXO03_viral
GS669_GCVIDIU02_0p1
GS665_G8GNNDP_3p0
GS665_G8GNNDP_0p8
GS665_GCX5D7Y01_0p1
GS666_G9JOFJ3_3p0
GS666_G9JOFJ3_0p8
GS666_G8GNNDP_0p1
GS666_G8GNNDP_viral
GS673_G8TPZVL_3p0
GS673_G8TPZVL_0p8
GS673_G8TPZVL_0p1
GS674_HAVSY8S_3p0
GS674_HAVSY8S_0p8
GS674_G8TPZVL_0p1
GS659_G8KGLNH_3p0
GS659_G8KGLNH_0p8
GS659_GCX5D7Y01_0p1
GS660_G8GNNDP_3p0
GS660_G8KGLNH_0p8
GS660_G8KGLNH_0p1
GS660_G8KGLNH_viral
GS677_HAVSY8S_3p0
GS677_HAVSY8S_0p8
GS677_GCVEAXJ02_0p1
GS678_HA8TPRT_HAQD3ZU_3p0
GS678_HA8TPRT_HAQD3ZU_0p8
GS678_GCVEAXJ02_0p1
GS678_GLDFQNX01_viral
GS679_GDNEDKP02_3p0
GS679_HAQD3ZU_0p8
GS679_HAQD3ZU_0p1
GS679_GLDFQNX01_viral
GS680_GDNEDKP02_3p0
GS681_HAT060T_3p0
GS681_HA8TPRT_HAQD3ZU_0p8
GS681_GCXE2IL02_0p1
GS682_HAT060T_3p0
GS682_HAT060T_0p8
GS682_HAT060T_0p1
GS683_HAISPF3_3p0
GS683_HAISPF3_0p8
GS683_GCXE2IL02_0p1
GS684_HA07AON_3p0
GS684_HAISPF3_0p8
GS684_HAISPF3_0p1
GS685_HA07AON_3p0
GS685_HA07AON_0p8
GS685_GCZC3J301_0p1
GS686_HAOJ6US_3p0
GS686_HAOJ6US_0p8
GS686_HA07AON_0p1
GS694_HABNP3B_3p0
GS694_HABNP3B_0p8
GS694_HABNP3B_0p1
GS695_HABNP3B_3p0
GS695_HABNP3B_0p8
GS695_GDQ27C301_0p1
GS695_GLDFQNX02_viral
GS687_HAOJ6US_3p0
GS687_HAOJ6US_0p8
GS687_GCZC3J301_0p1
GS688_HAN8WWE_3p0
GS688_HAN8WWE_0p8
GS688_GCZC3J302_0p1
GS689_HAN8WWE_3p0
GS689_HAN8WWE_0p8
GS689_GCZC3J302_0p1
GS689_GQY5VXO04_viral
